# Role of Misfolded N-CoR Mediated Transcriptional Deregulation of Flt3 in Acute Monocytic Leukemia (AML)-M5 Subtype

**DOI:** 10.1371/journal.pone.0034501

**Published:** 2012-04-13

**Authors:** Dawn Sijin Nin, Wai Kay Kok, Feng Li, Shinichiro Takahashi, Wee Joo Chng, Matiullah Khan

**Affiliations:** 1 Cancer Science Institute, Yong Loo Lin School of Medicine, National University of Singapore, Singapore; 2 Departments of Medicine, Yong Loo Lin School of Medicine, National University of Singapore, Singapore; 3 Department of Hematology-Oncology, National Cancer Institute of Singapore, National University Health System, Singapore, Singapore; 4 Division of Hematology, Kitasato University School of Allied Health Science, Kanagawa, Japan; Université Paris-Diderot, France

## Abstract

The nuclear receptor co-repressor (N-CoR) is a key component of the generic multi-protein complex involved in transcriptional control. Flt3, a key regulator of hematopoietic cell growth, is frequently deregulated in AML (acute myeloid leukemia). Here, we report that loss of N-CoR-mediated transcriptional control of Flt3 due to misfolding, contributes to malignant growth in AML of the M5 subtype (AML-M5). An analysis of hematopoietic genes in AML cells led to the identification of Flt3 as a transcriptional target of N-CoR. Flt3 level was inversely related to N-CoR status in various leukemia cells. N-CoR was associated with the Flt3 promoter in-vivo, and a reporter driven by the Flt3 promoter was effectively repressed by N-CoR. Blocking N-CoR loss with Genistein; an inhibitor of N-CoR misfolding, significantly down-regulated Flt3 levels regardless of the Flt3 receptor mutational status and promoted the differentiation of AML-M5 cells. While stimulation of the Flt3 receptor with the Flt3 ligand triggered N-CoR loss, Flt3 antibody mediated blockade of Flt3 ligand-receptor binding led to N-CoR stabilization. Genetic ablation of N-CoR potentiated Flt3 ligand induced proliferation of BA/F3 cells. These findings suggest that N-CoR-induced repression of Flt3 might be crucial for limiting the contribution of the Flt3 signaling pathway on the growth potential of leukemic cells and its deregulation due to N-CoR loss in AML-M5, could contribute to malignant growth by conferring a proliferative advantage to the leukemic blasts. Therapeutic restoration of N-CoR function could thus be a useful approach in restricting the contribution of the Flt3 signaling pathway in AML-M5 pathogenesis.

## Introduction

Acute Monoblastic/Monocytic leukemia (AML-M5) is a class of Acute Myeloid Leukemia (AML) classified under the M5 subtype in the French-American-British (FAB) classification. It is defined as a group of malignant disorder characterized by the abnormal accumulation of immature cells of the myelo-monocytic lineage in the bone marrow and peripheral blood [1], [2] and constitutes about 5 to 10% of all AML cases in adult humans. Although the fusion oncogene MLL1-AF9 is mainly associated with AML-M5 [3], [4], it is not the only genetic anomaly present and other diverse genetic aberrations are also reported in the disease [5]. However, despite the varied genetic background of the disease, the phenotypic presentation is almost identical, characterized by the differentiation arrest at the monoblast and/or promonocytic stage coupled with increased survival and proliferation capacities: a hallmark of AMLs. Thus it is thought that aberrations involving key transcription factors and its associated co-activators and co-repressors essential for the differentiation process are major driving forces of AML-M5 pathogenesis.

One such factor is the nuclear receptor co-repressor (N-CoR), a key component of the multi-protein co-repressor complex involved in transcriptional repression mediated by various transcriptional factors. N-CoR was first identified as a co-repressor of un-liganded nuclear hormone receptors [6], [7] and was later demonstrated to be essential for the transcriptional repression mediated by Mad and other sequence-specific transcription factors [8], [9]. It was later identified as a Ski interacting protein in yeast two-hybrid assay [10] and was also demonstrated to have an essential role in the transcriptional repression of the tumor suppressors Mad and Rb [11], [12]. Our laboratory later reported that abrogation of N-CoR-mediated transcriptional repression due to a misfolded conformation dependent loss (MCDL) of N-CoR protein was associated with the differentiation arrest of leukemic cells in Acute Promyelocytic Leukemia (APL) [13], [14], [15]. Recently, N-CoR was also reported to be essential for the differentiation of erythroid cells [16]. These findings coupled with reports indicating that N-CoR knockout mice were embryonically lethal and appeared to die from anemia due to defects in definitive erythropoiesis [17], highlighted an essential role of N-CoR in the differentiation of cells during myeloid lineage commitment.

The cytokine receptor FMS-Like Tyrosine Kinase III (Flt3) is a membrane bound receptor tyrosine kinase (RTK) belonging to the RTK subclass III family, essential for normal hematopoiesis [18]. It is a key factor that maintains immature hematopoietic cells in an undifferentiated state by promoting their self-renewal and proliferative potentials [19], [20] and is expressed in majority of the human and mice repopulating hematopoietic stem cell (HSC) population [19], [21]. Involvement of Flt3 in the proliferation of HSCs and early progenitor cells suggests that Flt3 expression and activation of the Flt3 signaling pathway have possible oncogenic potentials. Evidence from clinical studies has indicated that Flt3 has the capacity to enhance survival and proliferation of leukemic blasts, with a high percentage of AMLs expressing Flt3 [22], [23], [24]. A contributing role of Flt3 in the transforming potential of PML-RARα and various MLL1 fusion proteins have been identified in several mice models of APL and AML-M5 [25], [26], [27], [28], [29], [30]. However the exact nature of this co-operation in the malignant growth and transformation of APL and AML-M5 cells is not known. Here we report that Flt3 (regardless of its mutational status) is a target of N-CoR mediated transcriptional repression and demonstrate how aberrant expression of the Flt3 receptor due to a post-translational loss of N-CoR contributes to the survival and growth advantage of leukemic cells in AML-M5. We also show that therapeutic restoration of N-CoR in AML-M5 cells may be a useful approach in restricting the role of Flt3 mediated survival and proliferative capacity in leukemic blasts.

## Materials and Methods

### AML cell lines, primary AML samples and reagent

The AML-M5 cell lines, THP-1, Mono-Mac-1 (MM1), Nomo-1, MV-4-11, non-AML-M5 cell lines U937, HL-60, K562 and APL cell line NB4 were maintained in RPMI 1640 medium (Life Technologies, Gaithersburg, MD) supplemented with 10% Fetal Bovine Serum (FBS; Hyclone Laboratories, Logan, UT), in a humidified atmosphere of 5% CO_2_. The AML-M5 cell line SigM5 was maintained in Isocove's modified medium (Life Technologies, Gaithersburg, MD) supplemented with 20% FBS while 293T cells were maintained in DMEM (Sigma Aldrich, MO, USA) enriched with 10% FBS. BA/F3 cells were maintained in RPMI 1640 medium supplemented with 10% FBS and 10 ng/ml recombinant mouse IL-3 (R&D systems, MN, USA). Cell lines were purchased from ATCC (Manassas, VA, USA), DSMZ - Deutsche Sammlung von Mikroorganismen und Zellkulturen GmbH (German Collection of Microorganisms and Cell Cultures, Germany) and Japan Health Sciences Foundation (Osaka, Japan).

Primary leukemic samples used in this study were obtained at the time of diagnosis. Diagnoses of AML were made from the morphology and cytochemistry according to the French–American–British (FAB) classification as well as immunophenotypic and cytogenetic analyses. This study was approved by the Institutional Review Boards of National University of Singapore. Informed consent was obtained from the patients in accordance with the Declaration of Helsinki.

The N-CoR [C-20] (goat polyclonal) antibody was purchased from Santa Cruz Biotechnology (California, USA) and used as described previously [14], [15]. Flt3 (C-20) and Flt3 (8H5) antibodies were from Santa Cruz Biotechnology (California, USA) while FITC-conjugated CD14 antibody was from BD Pharmingen (San Diego, CA, USA). N-CoR stabilizing agent Genistein (Sigma Aldrich, MO,USA) was used as previously described elsewhere [15].

### Real-time PCR assay

Total RNA was isolated using the RNeasy Mini Kit (Qiagen GmBH, Hilden, Germany). From each sample, 2 µg of RNA was converted into cDNA by oligo (dT)_18_-primed reverse transcription using SuperScript II RT First-Strand kit (Invitrogen, Carlsbad, CA, USA) as described by the manufacturer. First-strand cDNA was synthesized using SMART-PCR cDNA Synthesis Kit (Clontech). Real-time PCR analysis was carried out using the Taqman® Gene Expression Assay System (Applied Biosystems, CA, USA) and C_t_ values were recorded using the ABI Prism 7300 Real Time PCR system (Applied Biosystems, CA, USA).

### Analysis of Real-Time PCR data

For gene expression in cell lines, data was analyzed using the comparative C_t_ method where the cell line HL-60 was used as the reference sample and the HPRT gene was used as the endogenous gene control. Data representation for gene expression analysis is in the form of a bar graph plotted on a logarithmic scale with a base of 10, where expression level in the reference sample for all genes was set to 0 while genes which were up regulated relative to expression levels in the reference sample was given a positive value and those which are down regulated relative to expression levels in the reference sample was given a negative value. Raw C_t_ values that registered as undetermined were given a value of to 40 for the purpose of calculation and analysis. Data represented is the average obtained from 3 independent experiments.

### RT-PCR analysis

cDNA was subject to RT-PCR analysis using Accuprime Taq polymerase system (Invitrogen, Carlsbad, CA, USA) according to manufacturer's recommendations. The sequence of the primers is presented in Table S1.

### RNA interference

All siRNA (Qiagen, Hilden, Germany) were synthesized as fully annealed oligonucleotide duplexes. For siRNA-mediated knockdown of N-CoR in HL-60 cells, 2 µg of siRNA was transfected by electroporation using the Cell Line Nucleofector Kit V (Amaxa, Cologne, Germany) according to the manufacturer's optimized protocol. For 293T cells, siRNA was transfected using Lipofectamine 2000 (Invitrogen, Carlsbad, CA, USA). The siRNA target sequence for N-CoR knockdown is 5′AATGCTACTTCTCGAGGAAACA -3′. siRNA targeting the luciferase sequence 5′-CGTACGCGGAATACTTCGA-3′ was used as a control.

### Dual luciferase reporter assay

Leukemic cell lines HL-60, THP-1, K562 and U937 were co-transfected with 1 µg of Flt3 full-length promoter/firefly luciferase reporter plasmid or promoter-less pGL3-basic vector (23) and 5 ng of CMV/renilla luciferase plasmid by electroporation, using the Cell Line Nucleofector Kit (Amaxa, Cologne, Germany). The cells were harvested for luciferase assay, 48 hours post-electroporation, as described by the Dual Luciferase Assay Kit (Promega, WI, USA). 293T was co-transfected with 50 pmol of N-CoR-targeting siRNA, 1 µg of Flt3 full-length promoter/firefly luciferase reporter plasmid or promoter-less pGL3-basic vector, 5 ng of CMV/renilla luciferase plasmid and various dosages of pAct-Flag/N-CoR or its empty vector, using Lipofectamine 2000 (Invitrogen, Carlsbad, CA, USA). The cells were harvested and reporter activity determined 72 hrs post-transfection.

### ChIP assay

Chromatin Immunoprecipitation (ChIP) was carried out with the commercially available ChIP-IT kit (Active Motif, Carlsbad, CA, USA) according to the manufacturer's instructions. Prior to precipitation, an aliquot of the chromatin was taken as input DNA control. Chromatin linked to N-CoR was precipitated with either 3 µg of N-CoR [C-20] antibody (Santa Cruz Biotechnology, CA, USA) or 3 µg of normal goat IgG (Santa Cruz Biotechnology, CA, USA), as described by the kit's manual. The purified immunoprecipitated chromatin was subjected to RT-PCR analysis, using the Accuprime Taq polymerase system (Invitrogen, Carlsbad, CA, USA).

### Proliferation of BA/F3 cells after N-CoR knockdown

BA/F3 cells transfected with either 2 µg of N-CoR siRNA or 2 µg of control siRNA via electroporation using the Amaxa Cell line Nucleofector Kit V (Amaxa, Cologne, Germany). Cells were allowed to recover in IL-3 containing growth medium for 48 hrs to allow for Flt3 receptor expression. Cells were then washed in 1× PBS, and resuspended in IL-3 free culture medium or rm-Flt3 ligand (100 ng/ml) (R&D systems, MN, USA) supplemented media. Cell growth was analyzed using the Cell Proliferation Kit I [3-(4, 5-dimethylthiazol-2-yl)-2,5-diphenyltetrazolium bromide; (MTT)] (Roche, Germany) as described by the manufacturer. The spectrophotometric absorbance was measured using a microplate reader (Ultramark, Biorad, CA, USA) at wavelength 595 nm with a reference wavelength of 655 nm.

### N-CoR status in rh-Flt3 ligand stimulated hFlt3 receptor expressing 293T cells

293T cells were transfected with either 6 µg of MSCV-GFP-Flt3 (WT) expression vector or 6 µg MSCV-GFP-Empty vector and incubated for 24 hours. After which cells were serum starved overnight and stimulated with 30 ng/ml of rh-Flt3 ligand for 4 hours before cells are assayed for SDS-PAGE and Western Blotting Analysis.

### Stabilization of N-CoR in rh-Flt3 ligand stimulated THP-1 cells

THP-1 cells were serum starved overnight and seeded at a density of 4×10^5^ cells/ml in 3 mls of serum free media in a 6-well plate. Anti-Flt3 antibody or control IgG was added in various amounts (1, 0.5, 2.5,5 µg) and cells were incubated for 60 minutes at 37°C in a humidified atmosphere of 5% CO2. Cells were then stimulated with 30 ng/ml of rh-Flt3 ligand for 4 hours before harvesting for protein expression analysis.

### Cell Proliferation Assay

The cell proliferation assay was carried out using the Cell Proliferation Kit I [3-(4, 5-dimethylthiazol-2-yl)-2, 5-diphenyltetrazolium bromide; (MTT)] (Roche, Germany) as described by the manufacturer using cells treated at various concentrations of Genistein for the stipulated treatment durations. The spectrophotometric absorbance was measured using a microplate reader (Ultramark, Biorad, CA, USA) at wavelength 595 nm with a reference wavelength of 655 nm.

### Statistical analysis

The results of the proliferation assays were reported as mean ± SD. Statistical analysis was performed using unpaired t-test. P value less than 0.05 was considered to be statistically significant.

### Cell differentiation assay

THP-1 cells treated with various concentrations of Genistein or vehicle (DMSO) were collected and incubated with FITC-conjugated monoclonal mouse anti-human CD14 antibody or control IgG (Pharmingen, San Diego, CA) as per manufacturer's protocol. Antibody conjugated cells were analyzed using Fluorescence Activated Cell Sorting (NUMI core facility, National University of Singapore).

For morphological analysis of THP-1 cells treated with Genistein, cells were cytospun onto slides and stained with Wright-Giemsa Stain and examined under light-microscopy.

## Results

### N-CoR loss correlates with the up-regulation of Flt3 expression

Previously our laboratory reported the role of N-CoR loss in the pathogenesis of APL and restoration of N-CoR function via Genistein, a tyrosine kinase inhibitor isolated from soy relieves the block in differentiation and ultimately induced cell death [14], [15]. Recently, we have also identified a similar APL-like post-translational N-CoR loss in Acute Monocytic Leukemia (AML of the M5 subtype in the French-American-British classification-AML-M5). Given N-CoR's documented importance in hematopoiesis and its role as a transcriptional co-repressor, we hypothesized that N-CoR loss in AML-M5 cells may have altered the expression profile of genes associated with the normal growth and maturation of hematopoietic cells, eventually contributing to malignant transformation. We therefore decided to identify the hematopoietic genes which expressions could be affected by the loss of N-CoR in AML-M5 cells.

**Figure 1:**
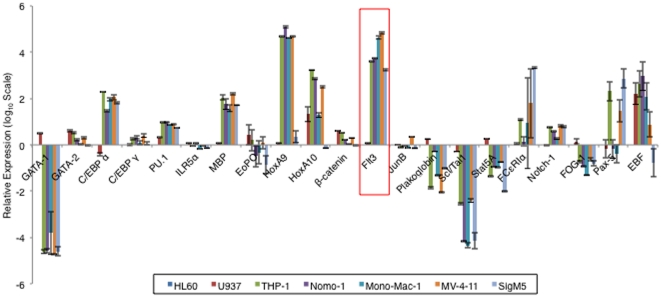
N-CoR loss is associated with the up-regulation of Flt3. *A*, Relative expressions of selected hematopoietic genes in AML-M5 and non-AML-M5 (HL-60 and U937) cells were determined by real time PCR analysis. Data was analyzed via the comparative C_t_ method with the expression level of each gene in HL-60 cells set as the reference value, and the level of expression of the HPRT gene used as the endogenous control. The graph was plotted on a logarithmic scale with a base of 10. Expression levels in HL-60 cells for all genes were set to 0 while genes which were up-regulated relative to their expression levels in HL-60 cells was given a positive value, and those which were down-regulated relative to expression level in HL-60 cells were given a negative value. Raw C_t_ values that registered as undetermined were given a value of 40. (Results presented are the averages of 3 independent experiments.).

**Figure 2:**
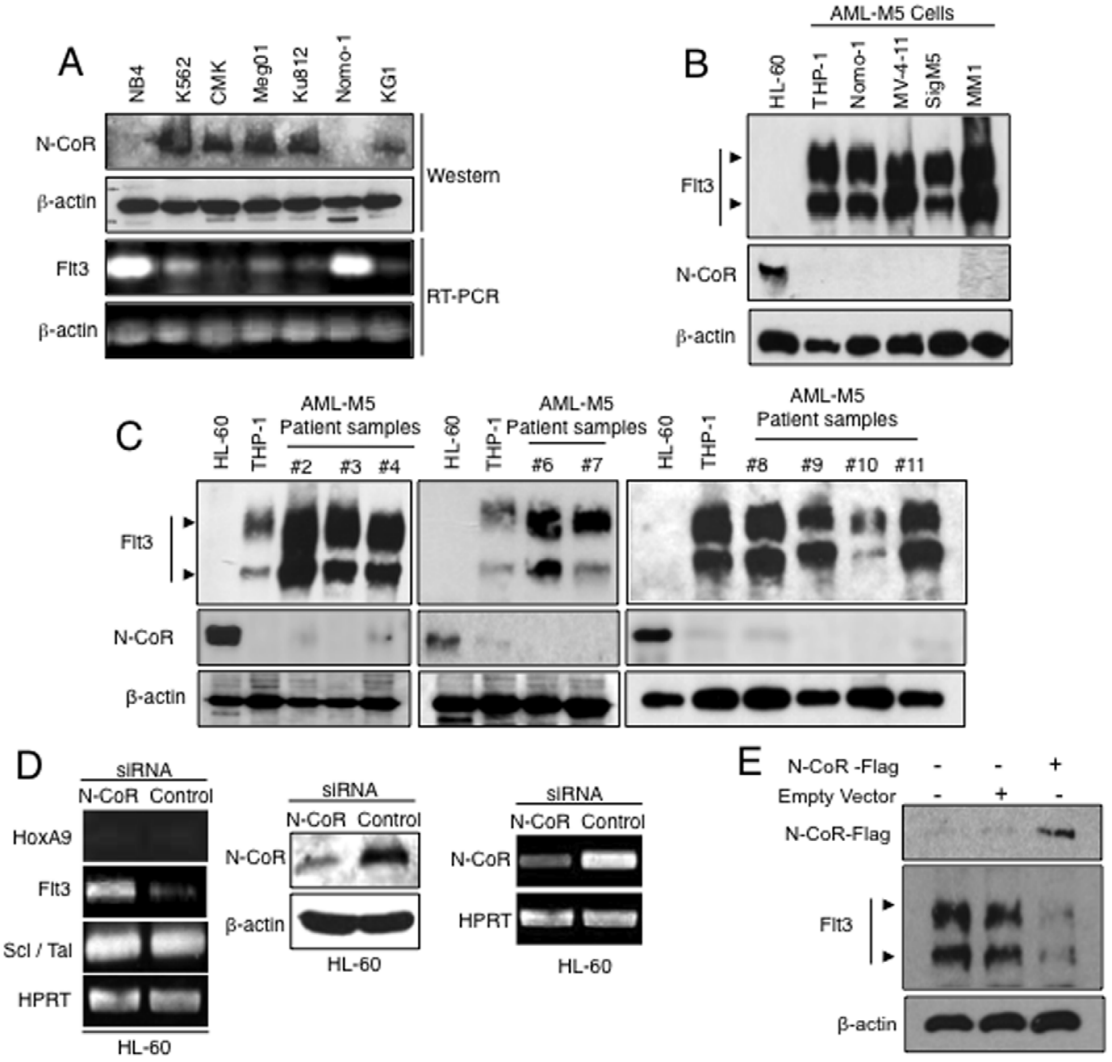
Flt3 expression is inversely related to N-CoR protein status. *A*, N-CoR and Flt3 levels in various human leukemia cells were determined in western blotting and RT-PCR analysis respectively. *B & C*, Flt3 and N-CoR levels in AML-M5 derived cell lines (*B*) and in multiple histologically confirmed human primary AML-M5 samples (*C*) were determined through western blotting assay using the respective antibodies. Levels of N-CoR and Flt3 in HL-60 cells were used as a reference. *D*, Levels of Flt3, HoxA9 and Scl/Tal in HL-60 cells transfected with N-CoR or control siRNA were determined by RT-PCR analysis (left panel). N-CoR knockdown efficiency at protein (middle panel) and transcript level (right panel) in HL-60 cells transfected with N-CoR or control siRNA was determined through western blotting assay and RT-PCR analysis. *E*, Ectopic expression of Flag-tagged N-CoR in THP-1 cells resulted in the loss of Flt3 expression as determine via western blotting assay with anti-Flt3 antibody. Levels of ectopic N-CoR expression were determined via western blotting assay with anti-Flag antibody.

Comparative Real-Time PCR analysis of 21 hematopoietic genes [31] in 2 subsets of AML cells, the N-CoR positive cells, HL-60 (a AML-M2 derived cell line) and U937 (a monocytic cell line derived from histocystic lymphoma), and the 5 AML-M5 cells namely THP-1, Nomo-1, Mono-Mac-1 (MM1), MV-4-11 and SigM5 in which N-CoR was lost, identified Flt3 as the gene selectively up-regulated in all AML-M5 cells (Fig. 1 and Fig. S1). Analysis of more N-CoR positive and negative cells lines (Fig. 2A) further established the inverse correlation between N-CoR status and the level of Flt3 gene expression. The inverse correlation between N-CoR and Flt3 expression was also found to be translated to the protein level in the AML-M5 cell lines (Fig. 2B) as well as in multiple histologically confirmed primary AML-M5 patient samples (Fig. 2C), where both the 130 kDa intracellular non–glycosylated and the 160 kDa membrane bound glycosylated forms were observed to be highly expressed. Furthermore, siRNA mediated N-CoR knockdown performed on N-CoR positive HL-60 revealed that after N-CoR ablation, Flt3 transcript levels was significantly up-regulated while the levels of two other genes which did not have a correlation with N-CoR status was not altered (Fig. 2D left panel). Successful N-CoR knockdown in HL-60 cells was determined via western blotting and RT- PCR (Fig. 2D middle and right panel). Conversely, over-expression of Flag- tagged N-CoR in THP-1 cells brought about a down-regulation of Flt3 levels (Fig. 2E).

### Flt3 is a transcriptional target of N-CoR

**Figure 3:**
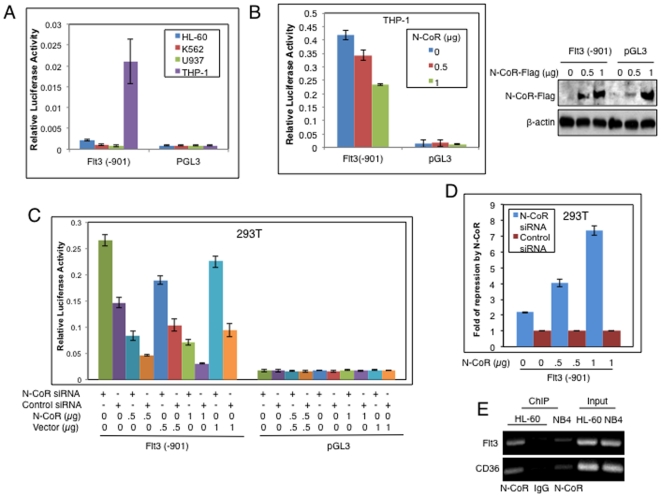
Repression of Flt3 by N-CoR protein. *A*, Relative activity of a luciferase reporter driven by the Flt3 promoter was determined in various leukemic cell lines. The cells were transfected with reporter and reference plasmids using electroporation. The values presented in each bar represent the average of three independent experiments. *B*, Effect of ectopic N-CoR on the activity of the Flt3 promoter in THP-1 cells electroporated with Flag-tagged N-CoR plasmid in a dose dependent manner was determined via luciferase assay (left panel). The values presented in each bar represent the average of three independent experiments. In parallel, levels of ectopic N-CoR protein in THP-1 cells used in the luciferase assay were determined in western blotting assay with anti-Flag antibody (right panel). *C*, Effect of ectopic N-CoR on the activity of the Flt3 promoter in 293T cells transfected with N-CoR or control siRNA was determined using luciferase assay. In pGL3-Flt3 (−901) reporter plasmid, luciferase reporter was placed under the control of the full length Flt3 promoter. The values presented in each bar represent the average of three independent experiments. *D*, The dose dependent fold repression by ectopic N-CoR in N-CoR ablated or non-ablated 293T cells was calculated by dividing the mean relative luciferase activity in N-CoR siRNA transfected cells with that of control siRNA transfected cells of Fig. 3C. *E*, N-CoR is associated with the Flt3 promoter. Relative amounts of Flt3 promoter sequence associated with N-CoR protein in HL-60 or NB4 cells were determined through ChIP assay. The antibody used in the ChIP assay is mentioned at the bottom. N-CoR association with CD36 promoter, a known N-CoR target gene, was determined as positive control.

The inverse correlation between N-CoR and Flt3 expressions suggested that the reduced Flt3 levels in cells which expressed intact N-CoR protein might have resulted from a direct repression of this gene by N-CoR. Therefore, to show that N-CoR was indeed involved in the repression of Flt3, the activity of a luciferase reporter driven by the full length Flt3 promoter was compared in N-CoR positive and negative leukemic cells. The Flt3-luciferase reporter activity was significantly lower in N-CoR intact HL-60, K562 and U937 cells whereas in THP-1 cells, which lacked an intact N-CoR protein, reporter activity was significantly higher (Fig. 3A). Introduction of ectopic N-CoR in THP-1 cells (Fig. 3B, left panel) resulted in a dose dependent reduction of Flt3 promoter activity (Fig. 3B, right panel). To further prove that the Flt3 promoter was repressed by N-CoR, the effect of ectopic N-CoR expression on Flt3 promoter activity was determined via luciferase assay performed in 293T cells. In the initial experiments, it was noted that despite repeated attempts, no significant reduction in the Flt3 reporter activity by ectopic N-CoR was observed in 293T cells (data not shown). Thinking that this lack of reduction in the Flt3 reporter activity by ectopic N-CoR could be a result of the high levels of endogenous N-CoR protein present in 293T cells, the experiment was next repeated using N-CoR ablated 293T cells. N-CoR ablation by N-CoR siRNA (Fig. S2) increased the basal Flt3 reporter activity in 293T cells when compared to its activity in non-ablated cells (Fig. 3C). Moreover, ectopic restoration of N-CoR in N-CoR ablated 293T cells down-regulated this augmented Flt3 promoter activity in a dose dependent manner, and its value came down to a level that was lower than the basal value (Fig. 3C). The degree of Flt3 promoter inhibition by ectopic N-CoR was only two fold in N-CoR intact 293T cells irrespective of dose; while in N-CoR ablated cells, a seven fold reduction was observed when N-CoR was introduced at a maximum concentration of 1 µg (Fig. 3D). These observations suggest an active role of N-CoR in the repression of the Flt3 promoter. Next, to validate that N-CoR suppression of Flt3 expression was via its binding to the Flt3 promoter region, association of N-CoR protein with the promoter of Flt3 was analyzed by chromatin immunoprecipitation (ChIP) assay. To map the region of the Flt3 promoter specifically associated with N-CoR, chromatin extracts of HL-60 or NB4 cells were immunoprecipitated with anti-N-CoR antibody. The DNA co-precipitated with N-CoR was amplified with primers flanking various regions of the Flt3 promoter. Primers were designed to amplify DNA in each of the following regions within the Flt3 promoter sequence, more than 901 bps, between 614 bps to 814 bps and between 75 bps to 272 bps, upstream of the transcriptional start site. Only the primers located in the 614 bps to 814 bps region upstream of the transcriptional start site in the Flt3 promoter region (Fig. S3) was able to amplify the DNA co-precipitated with N-CoR antibody, suggesting that N-CoR and its associated repressor complex may have bound to the Flt3 promoter through a region located in that particular part of the promoter sequence (Fig. 3E). CD36, a known N-CoR target gene, was used as a positive control in this assay. Taken together, the data obtained so far indicates that Flt3 repression may be brought about by the recruitment of the N-CoR repressor complex to its promoter region and loss of N-CoR in AML-M5 results in the subsequent loss of Flt3 gene repression in these cells.

### N-CoR loss promotes the IL-3 independent growth potential of BA/F3 cells via the up-regulation of Flt3

**Figure 4:**
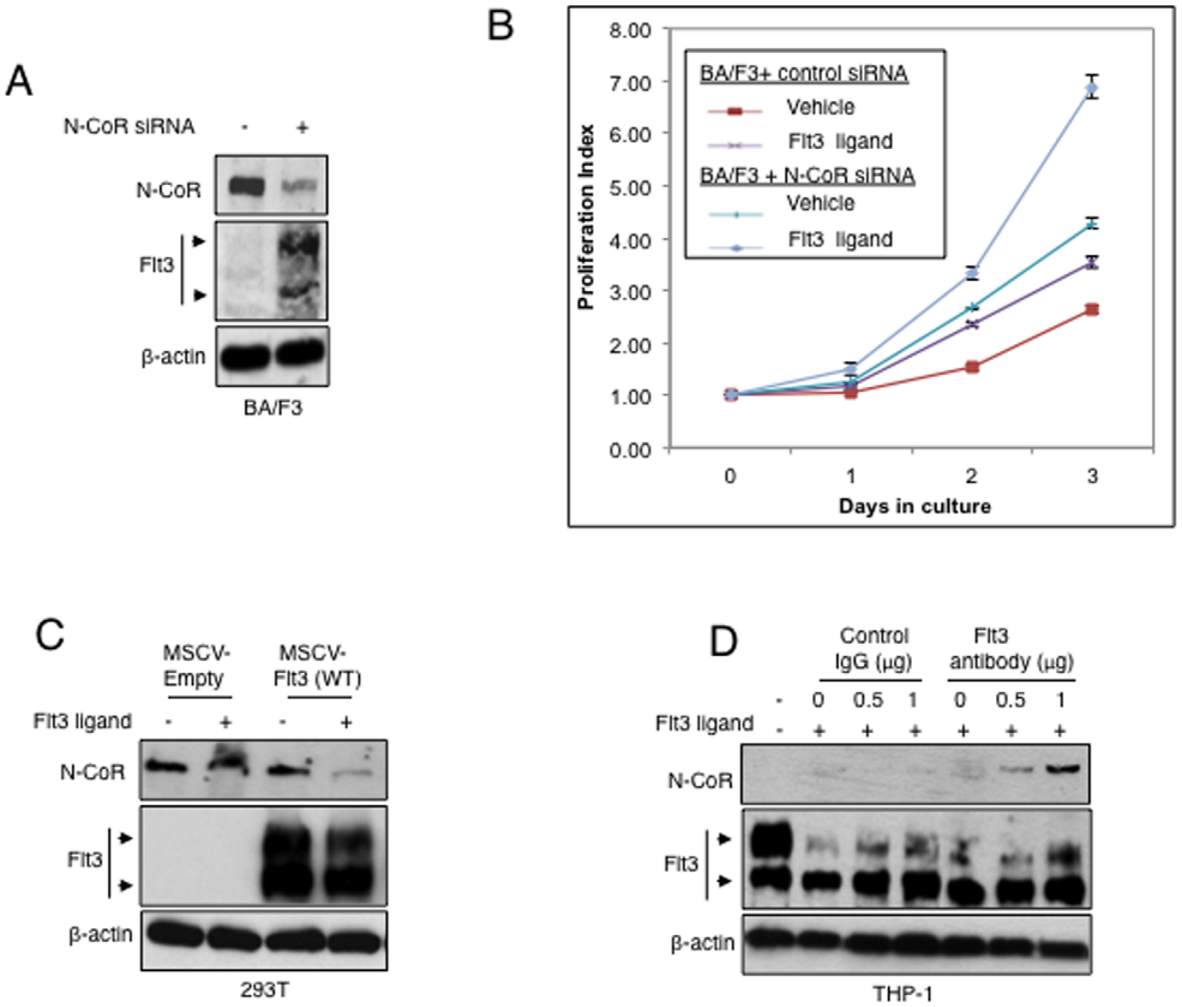
N-CoR loss promotes growth potential, which is amplified by Flt3 signaling activation. *A*, Levels of N-CoR and Flt3 protein in Ba/F3 cells transfected with control or N-CoR siRNA were determined via western blotting assay with an anti-Flt3 antibody that recognizes the murine form of the receptor and anti-N-CoR antibody. *B*, N-CoR loss mediated Flt3 expression enhanced the IL-3 independent growth potential of BA/F3 cells. Effect of IL-3 independent growth of Ba/F3 cells transfected with control or N-CoR siRNA (population from 4A) with and without Flt3 ligand stimulation were determined by cell proliferation assay. The Y-axis of the graph represents the proliferation index of viable cells, and the duration of culture is plotted on the X-axis. The symbols used in the graph are as follows: Control siRNA transfected cells treated with vehicle (<$>\raster="rg1"<$>), Control siRNA transfected cells stimulated with Flt3 ligand (<$>\raster="rg2"<$>), N-CoR siRNA transfected cells treated with vehicle (<$>\raster="rg3"<$>) and N-CoR siRNA transfected cells stimulated with Flt3 ligand (<$>\raster="rg4"<$>). The values presented in each graph are average of three independent experiments. *C*, Flt3 stimulation leads to N-CoR loss. Levels of N-CoR and Flt3 proteins in 293T cells treated with vehicle or Flt3 ligand (30 ng/ml) was determined by western blotting assay. *D*, Blocking Flt3 stimulation leads to N-CoR stabilization in THP-1 cells. Effect of Flt3 antibody on the levels of N-CoR and Flt3 proteins in THP-1 cells treated with vehicle or Flt3 ligand (30 ng/ml) was determined by western blotting assay.

Given the importance of Flt3 in the maintenance of survival and proliferative capabilities of HSCs as well as leukemic blasts, we hypothesized that aberrant expression of the receptor due to N-CoR loss may be crucial in providing AML-M5 cells with a survival and proliferative advantage. Thus in a proof of concept approach, we utilized BA/F3 cells, an IL-3 dependent murine bone marrow-derived cell line which expressed undetectable levels of the Flt3 receptor and a high level of endogenous N-CoR in an attempt to analyze the effects of N-CoR loss and Flt3 expression, on the IL-3 independent growth properties of these cells. First N-CoR levels were ablated in BA/F3 cells via siRNA mediated gene knockdown and levels of Flt3 protein expression assessed via western blotting with an antibody which was capable of detecting the murine form of the Flt3 receptor. It was observed that in N-CoR ablated BA/F3 cells; there was an increase in the level of expression of the Flt3 protein (Fig. 4A). Next, we looked at the IL-3 independent growth properties of these N-CoR intact and N-CoR ablated BA/F3 cells, in the presence and absence of the Flt3 ligand. It was observed that in the absence of IL-3, N-CoR ablated BA/F3 cells had a slight proliferative advantage over non-ablated cells. When stimulated with the Flt3 ligand, this proliferative advantage was enhanced 2 fold. This suggests that N-CoR loss promoted the IL-3 independent growth potential of BA/F3 cells and this growth capacity could be potentiated by the activation of the Flt3 signaling pathway via Flt3 ligand simulation (Fig. 4B).

### N-CoR loss is potentiated by Flt3 signaling activation

With the observations in BA/F3 cells and our recent findings which linked Akt activity to N-CoR loss (Nin et al, submitted manuscript), we hypothesized that Flt3 activation by the Flt3 ligand might have potentiated the effects of N-CoR loss, thus amplifying the growth advantage in AML-M5. To test this hypothesis, we first tested the effect of Flt3 ligand stimulation on the level of N-CoR protein in 293T cells in the presence or absence of the Flt3 receptor. As shown in Figure 4C, Flt3 ligand down-regulated N-CoR protein level in 293T cells in a Flt3 receptor dependent manner. To investigate if N-CoR loss in AML-M5 cells was potentiated by Flt3 activation, we looked at the effects of blocking Flt3 ligand-receptor interaction on the status of N-CoR in THP-1 cells. Selective blockade of Flt3 ligand-receptor binding with anti-Flt3 antibody led to the stabilization of N-CoR protein in THP-1 cells in a dose dependent manner (Fig. 4D). These data suggest that an oncogenic stimulus, which promotes cellular growth through Flt3 activation could amplify Flt3 mediated survival by, further inducing N-CoR loss and receptor expression up-regulation.

### Restoration of N-CoR function by Genistein, down-regulates Flt3, inhibits cell growth and induces terminal differentiation of AML-M5 cells regardless of Flt3 receptor mutational status

**Figure 5:**
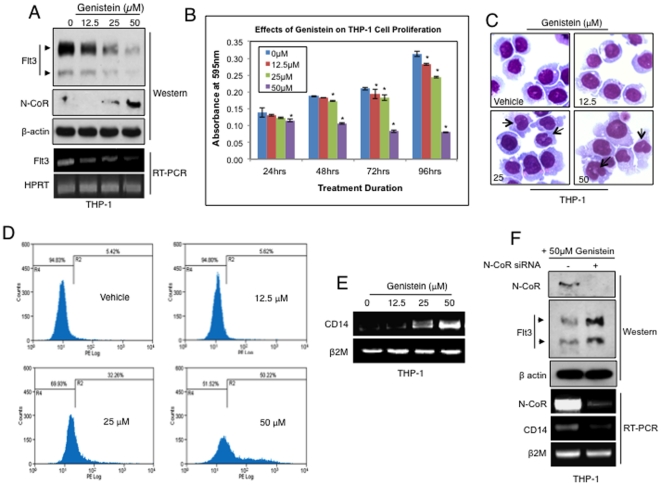
Restoration of N-CoR function in THP-1 cells, down-regulated Flt3 levels and induced differentiation. *A*, Flt3 levels were down-regulated at both the protein and mRNA levels after Genistein treatment. Level of Flt3 expression was determined via western blotting and RT-PCR analysis. *B*, Genistein inhibited the proliferation of THP-1 cells in a dose dependent manner when determined via growth proliferation assay (MTT). Results are representative of 3 independent experiments and asterisks represent p<0.05. *C*, Nuclear morphology of THP-1 cells treated with Genistein for the duration of 72 hours in a dose dependent manner was determined in Wright–Giemsa assay. The arrowheads mark the indented-shaped nucleus of differentiated cells. *D* & *E*, CD14 levels in THP-1 cells treated with Genistein for 72 hours in a dose dependent manner was determined by FACS (*D*) and RT-PCR (*E*) analysis. *F*, N-CoR, Flt3 and CD14 levels in THP-1 cells transfected with N-CoR siRNA and treated with 50 µM Genistein for 72 hours was determined by western blotting assay and RT-PCR analysis.

Next, we decided to look at how the restoration of N-CoR function in AML-M5 cells could affect the growth and proliferative properties of AML-M5 cells expressing the Flt3 wild type and/or mutant receptors. Utilizing Genistein, a drug which we have previously shown to restore N-CoR native properties in APL [15] and AML-M5 cells (Nin et al, submitted manuscript), we first assessed the effect of Genistein induced stabilization of N-CoR on the restoration of its repressive function on wild type Flt3 in the AML-M5 cell line THP-1 (a cell line reported to express the wild type Flt3 receptor). It was observed that in THP-1 cells, Genistein down-regulated Flt3 expression at both the transcript and protein levels with maximum loss of expression occurring at 50 µM, the dose which most effectively restored N-CoR protein expression (Fig. 5A). Next we looked at the effect of N-CoR expression and function restoration on the proliferative properties of THP-1 cells via MTT assay. We noticed a dose dependent inhibition of the growth capacity of treated cells. This inhibition was most pronounced again at the dose of 50 µM (Fig. 5B). Morphological analysis via Wright-Giemsa Staining of treated cells, revealed that this growth inhibition was likely due to the relieve of differentiation arrest as a significant number of Genistein-treated THP-1 cells displayed characteristics of matured monocytic cells such as horseshoe-shaped nuclei (Fig. 5C). Genistein induced differentiation progression was further supported by the ability of Genistein to up-regulate the level of CD14, a marker for myeloid/monocytic lineage maturation in THP-1 cells in a dose dependent manner when analyzed by FACS and RT-PCR analysis (Fig. 5D and E). This suggested that Genistein induced growth arrest in THP-1 cells was likely due to the restoration of the ability of these cells to progress in myeloid/monocytic lineage differentiation. Next, in order to determine if the restoration of N-CoR function had a role in this progression in differentiation, we proceeded to knockdown N-CoR via siRNA mediated gene knockdown in THP-1 cells and looked at the ability of these cells to differentiate after Genistein treatment. The expression level of the monocytic cell maturation marker CD14 at the transcript level was used as an indicator for progression of differentiation. It was observed that in the cells where N-CoR was knocked down, there was no restoration of N-CoR protein expression and function as Flt3 levels were not reduced compared to the non-ablated cells. CD14 transcript levels after Genistein treatment was also not significantly induced in the N-CoR ablated cells (Fig. 5F), indicating that N-CoR function was necessary in Genistein induced Flt3 down-regulation as well as the growth inhibition and differentiation progression of THP-1 cells.

**Figure 6:**
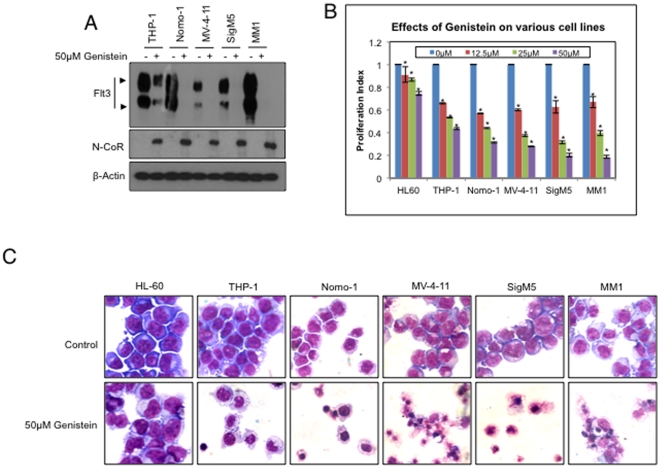
Restoration of N-CoR function attenuates the proliferative properties of AML-M5 cells regardless of the Flt3 receptor mutational status. *A*, Genistein stabilized N-CoR in all 5 AML-M5 cell lines at 50 µM with a concurrent down-regulation of the Flt3 receptor expression as determined via western blotting. *B*, Restoration of N-CoR function in AML-M5 cells attenuated the proliferative potential of AML-M5 cells. Proliferative capacity of N-CoR positive AML cells HL-60 and N-CoR negative AML-M5 cells after treatment with Genistein at 72 hours was determined via growth proliferation assay (MTT). Growth inhibition was most pronounced at 50 µM in AML-M5 cells while at the same dose, growth inhibitory effects on N-CoR positive HL-60 was less pronounced. Results are representative of 3 independent experiments and asterisks represent p<0.05. *C*, Morphology of N-CoR positive HL-60 and N-CoR null AML-M5 cells (THP-1, Nomo-1, MV-4-11, SigM5, MM1) as determined via Wright-Giemsa staining after treatment with 50 µM Genistein for 72 hours.

Next to investigate the effects of N-CoR function restoration on the proliferative properties of AML-M5 cells expressing the various mutants of the Flt3 receptor, the status of N-CoR after Genistein treatment in a panel of AML-M5 cells (THP-1 and Nomo-1- both expressing wild type Flt3, MM1- expressing a constitutively active Flt3-TKD mutant due to an activating point mutation at position 592 in the kinase domain, MV-4-11-expressing the constitutively active Flt3-ITD mutant receptor and Sig M5-no published reports on the status of the Flt3 receptor) was assessed. After treatment with Genistein at 50 µM concentration, it was observed that full length N-CoR was stabilized in all 5 AML-M5 derived cell lines and this was accompanied by the observable down-regulation of the Flt3 receptor expression regardless of the mutational status of the receptor in these cells (Fig. 6A). Next, the effect of N-CoR function restoration on the proliferative properties of these AML-M5 cells was investigated. It was noted that Genistein inhibited the proliferative capacity of all the N-CoR negative AML-M5 cells at the effective dose of 50 µM while its growth inhibitory effect on the N-CoR positive HL-60 cells was less pronounced (Fig. 6B and C). Together, these observations suggest that restoration of N-CoR function may be a potential therapeutic strategy in restricting the contribution of the Flt3 signaling pathway on the growth and proliferative properties of AML-M5 leukemic blasts independent of the Flt3 receptor mutational status.

## Discussion

Our laboratory had previously reported the role of post-translational N-CoR loss in the pathogenesis of APL [13], [14], [15]. The work presented in this manuscript was aimed at identifying how the APL-like post-translational loss of N-CoR could contribute to the pathogenesis of AML-M5 and to illustrate the potential of N-CoR as a novel molecular target in AML-M5 therapy. Here we reported that Flt3 is a target of N-CoR mediated transcriptional control. When this control is abolished due to post-translational N-CoR loss, AML-M5 cells could reacquire the capacities for growth and survival, which ultimately leads to leukemogenesis in conjunction with other factors involved in differentiation arrest. In normal cells, Flt3 is involved in the growth of early progenitor cells and recent studies by Kikushige et al reported that Flt3 expression in human HSCs, Granulocyte/Macrophage Progenitor stages promoted and maintained cell survival in these cells. This suggests that Flt3 has a critical role in the survival of the stem and progenitor cells and present as important targets for AML transformation [32]. N-CoR acts as a co-repressor for various transcriptional factors and is not known to bind directly to any specific DNA sequence. Therefore, it is not clear if its association with the Flt3 promoter is direct, or mediated indirectly by other DNA binding transcription factors. Although the transcription factor binding sites at the proximal part of the Flt3 promoter has been reported, there have been no studies suggesting the existence of any similar sites in its distal and middle regions. In the ChIP assay performed in this study, N-CoR was found to be specifically associated with the 614 bps to 814 bps upstream region of the transcriptional start site of the Flt3 promoter (Fig. S3). Identification of the factor that tethers N-CoR to the Flt3 promoter through this particular region might be crucial for the complete understanding of N-CoR's role in Flt3 signaling and its implication leukemogenesis.

Although Flt3 activating mutations have been widely associated with the poorer prognosis of AML patients, the fact that more than 70% of AMLs express wild-type Flt3 [24], [33], [34], implies that the native receptor is also important in the enhancement of survival and proliferation of leukemic blasts. In our study, we found that N-CoR regulates the expression of both the wild-type (THP-1 and Nomo-1) as well as the activating mutants of Flt3 (MV-4-11: FLT3-ITD, MM1: FLT3-TKDat position 592) as N-CoR loss and reciprocal up-regulation of Flt3 gene expression was found uniformly across all the AML-M5 cell lines used (There are no known reports on the status of the Flt3 receptor in SigM5). We also showed that knockdown of N-CoR in Ba/F3 cells resulted in the aberrant expression of Flt3 and conferred a proliferative advantage to Ba/F3 cells in IL-3 deficient conditions. With the activation of the Flt3 signaling pathway in the presence of activating factors, this IL-3 independent growth advantage was enhanced, suggesting that the loss of N-CoR mediated Flt3 repression in AML-M5 may have resulted in the aberrant expression of Flt3 and could enhance the survival and proliferation of AML-M5 blasts in the presence of factors or mutations which activate the Flt3 signaling pathway. Current therapies for AML-M5 in clinical practice include aggressive multi-drug chemotherapy, radiotherapy and allogenic bone marrow transplantations. However these current strategies have severe side effects with high morbidity rates. Flt3 inhibitors which target aberrant Flt3 activation in AMLs carrying the mutated Flt3 receptor have recently gained prominence but their effectiveness on the wild-type receptor is met with less success [35]; furthermore, resistance to Flt3 inhibitors is an emerging drawback of Flt3 inhibitor based therapy [36]. As many AMLs have been reported to express both mutant and wild type Flt3 receptors, targeting a common factor like N-CoR, which affects the expression of both receptors, could present as a useful and novel therapeutic approach in AML-M5 treatment to address these drawbacks. Our laboratory has recently identified various agents such as Genistein and Curcumin which effectively target N-CoR loss in APL [15], [37]. In this study, we have shown that restoration of N-CoR function by small molecules such as Genistein in AML-M5 cells effectively down-regulated Flt3 expression and reduced the growth capacity of these cells via the induction of terminal differentiation regardless of the Flt3 receptor mutational status. Recently, the molecular mechanism which mediates N-CoR loss in AML-M5 have been elucidated in our laboratory (Nin et al, submitted manuscript) and we have also identified several small molecules which were able to target this mechanism to restore N-CoR function and inhibit cellular growth in AML-M5, suggesting that N-CoR could pose as a plausible candidate for therapeutic targeting in AML-M5 independent of the Flt3 receptor status.

## Supporting Information

Figure S1 Relative expression of N-CoR protein in HL60, NB4 and THP-1 as determined via western blotting assay using anti-N-CoR antibody (left panel). RT-PCR analysis of selected hematopoietic genes in AML-M5, APL and N-CoR expressing HL-60 cells. Only the Flt3 gene expression showed an inverse relationship to N-CoR protein status in the cell lines used (right panel).
(TIFF)

Figure S2 N-CoR knockdown efficiency in experiments performed in Fig. 3C was determined by RT-PCR. All siRNA, from Qiagen, were synthesized as fully annealed oligonucleotide duplexes. The lyophilized siRNA were processed as described by the company's instructions before used. For siRNA-mediated knockdown of in 293T cells, siRNA was transfected into the cells using Lipofectamine 2000 (Invitrogen) as described by the manufacturer. The target sequence of siRNA used to knockdown N-CoR was 5′-AATGCTACTTCTCGAGGAAACA-3′. A mock siRNA targeting the luciferase sequence 5′-CGTACGCGGAATACTTCGA-3′, not found in the mammalian genome, was used as a non-specific control. 293T cell were transfected in 6-wells plates with 50 pmol of each type of siRNA. 293T were harvested 72 hours post-transfection for verification of knockdown efficiency by RT-PCT.
(TIFF)

Figure S3 Flt3 promoter sequence and ChIP primer priming site. The Flt3 promoter sequence up to −901 base pairs upstream of the transcriptional start site in exon 1(highlighted). The putative N-CoR binding region of the Flt3 promoter pulled down in ChIP assay is marked by a red box. The forward and reverse primers are indicated in bold font and grey highlights. The primers prime in a region upstream of known transcription factor binding sites that are indicated in black boxes. (Adapted from M.Inomata et al. Leukemia Research 30 (2006) 659–664).
(TIFF)

Table S1 List of RT-PCR primers used in this study.
(DOC)
