# Supplementary figures and images for: Role of Misfolded N-CoR Mediated Transcriptional Deregulation of Flt3 in Acute Monocytic Leukemia (AML)-M5 Subtype
Source: PLoS One. 2012 Apr 13;7(4):e34501. doi: 10.1371/journal.pone.0034501 (PMC3326026; doi:10.1371/journal.pone.0034501)

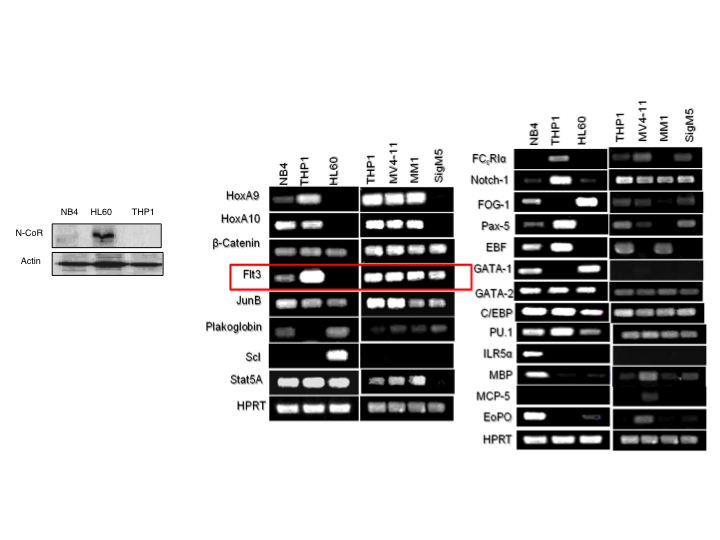

Supplement: Figure S1 — Relative expression of N-CoR protein in HL60, NB4 and THP-1 as determined via western blotting assay using anti-N-CoR antibody (left panel). RT-PCR analysis of selected hematopoietic genes in AML-M5, APL and N-CoR expressing HL-60 cells. Only the Flt3 gene expression showed an inverse relationship to N-CoR protein status in the cell lines used (right panel). (TIFF) [file pone.0034501.s001.tiff]

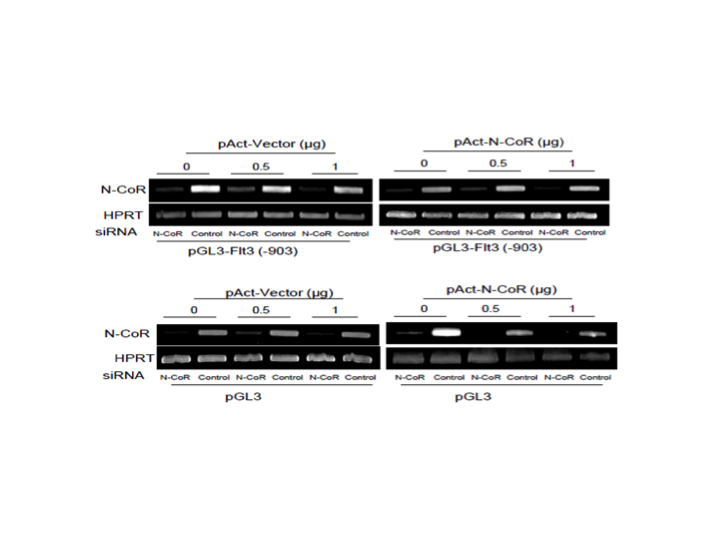

Supplement: Figure S2 — N-CoR knockdown efficiency in experiments performed in Fig. 3C was determined by RT-PCR. All siRNA, from Qiagen, were synthesized as fully annealed oligonucleotide duplexes. The lyophilized siRNA were processed as described by the company's instructions before used. For siRNA-mediated knockdown of in 293T cells, siRNA was transfected into the cells using Lipofectamine 2000 (Invitrogen) as described by the manufacturer. The target sequence of siRNA used to knockdown N-CoR was 5′-AATGCTACTTCTCGAGGAAACA-3′. A mock siRNA targeting the luciferase sequence 5′-CGTACGCGGAATACTTCGA-3′, not found in the mammalian genome, was used as a non-specific control. 293T cell were transfected in 6-wells plates with 50 pmol of each type of siRNA. 293T were harvested 72 hours post-transfection for verification of knockdown efficiency by RT-PCT. (TIFF) [file pone.0034501.s002.tiff]

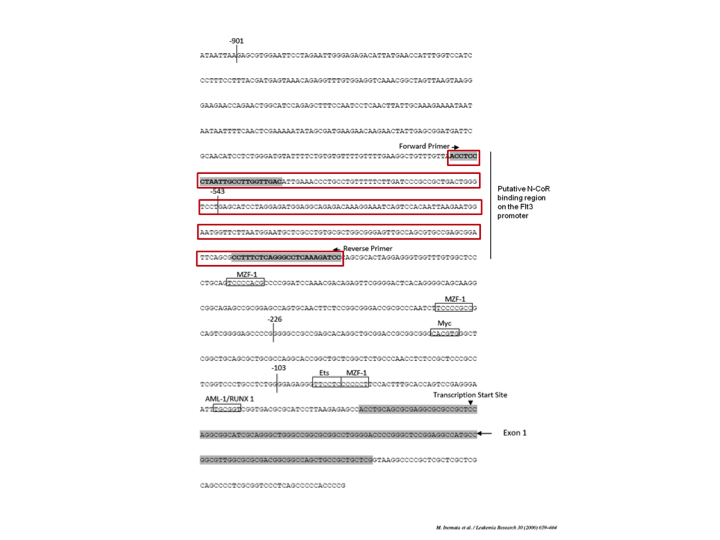

Supplement: Figure S3 — Flt3 promoter sequence and ChIP primer priming site. The Flt3 promoter sequence up to −901 base pairs upstream of the transcriptional start site in exon 1(highlighted). The putative N-CoR binding region of the Flt3 promoter pulled down in ChIP assay is marked by a red box. The forward and reverse primers are indicated in bold font and grey highlights. The primers prime in a region upstream of known transcription factor binding sites that are indicated in black boxes. (Adapted from M.Inomata et al. Leukemia Research 30 (2006) 659–664). (TIFF) [file pone.0034501.s003.tiff]
