# Supplementary material for: Role of Misfolded N-CoR Mediated Transcriptional Deregulation of Flt3 in Acute Monocytic Leukemia (AML)-M5 Subtype
Source: PLoS One. 2012 Apr 13;7(4):e34501. doi: 10.1371/journal.pone.0034501 (PMC3326026; doi:10.1371/journal.pone.0034501)
Supplement: Table S1 — List of RT-PCR primers used in this study. (DOC) [file pone.0034501.s004.doc]

**Supplemental Table S1:**

**Supplemental Table S1.** List of RT-PCR primers

| **Gene** | **Sequence** | **Annealing Temperature (^0^C)** | **Cycles** |
| --- | --- | --- | --- |
| GATA-1 | Forward: 5’-ATATGCCGGCTGGGCCTACG-3’  Reverse: 5’-GGTGGTCGTCTGGCAGTTGG-3’ | 60 | 30 |
| GATA-2 | Forward: 5’-TCATCTTCCGCGGGGGGTAG-3’  Reverse: 5’-GGACATCTTCCGGTTCCGAGTC-3’ | 60 | 30 |
| C/EBPα | Forward: 5’-CAAGCGGGTGGAACAGCTGAG-3’  Reverse: 5’-TGCTCCCCTCCTTCTCTCATGG-3’ | 60 | 30 |
| PU.1 | Forward: 5’-CGTGCACAGCGAGTTCGAGAG-3’  Reverse: 5’-GCGCGCCATCTTCTGGTAGG-3’ | 60 | 30 |
| IL5Rα | Forward: 5’-CCTGCAGAACGACCACTCACTAC-3’  Reverse: 5’-CACTCTCTCAAGGGCTTGTGTTC-3’ | 60 | 30 |
| MBP | Forward: 5’-GCGCTCAACCAGGGTCAAGTC-3’  Reverse: 5’-AAGAGAACTAGCTGAGCCCATTCC-3’ | 60 | 30 |
| MCP-5 | Forward: 5’-GCCTACCTGGAAATTGTAACTTCC-3’  Reverse: 5’-CAGCTGAAGATTGTGGTCAAAGTC-3’ | 60 | 35 |
| EoPO | Forward: 5’-TATGGCAGTGAGGTCTCCCTCTC-3’  Reverse: 5’-GGTACTGACTGTCCAAGCGGAAC-3’ | 60 | 30 |
| FCεRIα | Forward: 5’-GTGTTAGCAGTCCCTCAGAAACC-3’  Reverse:5’-TACAGTAATGTTGAGGGGCTCAG-3’ | 60 | 35 |
| Notch-1 | Forward: 5’-TACTACGGCCGCGAGGAGGA-3’  Reverse: 5’-TGGCAGACATGCGCAGGTCA-3’ | 60 | 30 |
| FOG-1 | Forward: 5’-CCTCCCAGCGCAGATGTTAACTC-3’  Reverse: 5’-GGTCTCTTTGGGCTTCTCGTCTG-3’ | 60 | 35 |
| PAX-5 | Forward: 5’-AGAGCGGGTGTGTGACAATGAC-3’  Reverse: 5’-GCACACTGCTCCCGATGTCAG-3’ | 60 | 35 |
| EBF | Forward: 5’-TGGCCCGGGCTCACTTTGAG-3’  Reverse: 5’-GAGCAAGACTCGGCACATTTCTG-3’ | 60 | 35 |
| HoxA9 | Forward: 5’-ATCCCAATAACCCAGCAGCCAAC-3’  Reverse: 5’-ACACACAGCTATCAGCACTAATGC-3’ | 60 | 30 |
| HoxA10 | Forward: 5’-GATTCCCTGGGCAATTCCAAAGG-3’  Reverse: 5’-CCCAGGAGATGGCGAGTGTG-3’ | 60 | 30/40 |
| β-catenin | Forward: 5’-GTAGAAGCTGGTGGAATGCAAGC-3’  Reverse: 5’-ATAGTGAAGGCGAACTGCATTCTGG-3’ | 60 | 30/40 |
| Flt3 | Forward: 5’-TCAGGGGCAATGCCCGTCTG-3’  Reverse: 5’-CTGCATCTGCCAGCTGACATCC-3’ | 60 | 30/40 |
| JunB | Forward: 5’-GCCTCCACCTTCAAGGAGGAAC-3’  Reverse: 5’-GGGCAGGGGACGTTCAGAAG-3’ | 60 | 30 |
| Plakoglobin | Forward: 5’-ACGAGGGCACTGCCACCTAC-3’  Reverse: 5’-AGGCCGTCGCTGTAGGTGTC-3’ | 60 | 30 |
| Scl/Tal1 | Forward: 5’-CTCGGCAGCGGGTTCTTTGG-3’  Reverse: 5’-CATTGAGCAGCTTGGCCAAGAAG-3’ | 60 | 30 |
| Stat5A | Forward: 5’-GGAAGTTTGACTCCCCGGAACG-3’  Reverse: 5’-CTGGCCACATCCATGGTCTCATC-3’ | 60 | 30 |
| HPRT | Forward: 5’-GAAGGAGATGGGAGGCCATCAC-3’  Reverse: 5’-CAACAATCCGCCCAAAGGGAAC-3’ | 60 | 30 |
